# Too much or too little opioids to patients receiving opioid agonist therapy in Norway (2013–2017): a prospective cohort study

**DOI:** 10.1186/s12913-020-05504-y

**Published:** 2020-07-20

**Authors:** Jørn Henrik Vold, Svetlana Skurtveit, Christer Aas, Kjell Arne Johansson, Lars Thore Fadnes

**Affiliations:** 1 Department of Addiction Medicine, Haukeland University Hospital, Bergen, Norway; 2 Department of Global Public Health and Primary Care, University of Bergen, Bergen, Norway; 3 Department of Mental Disorders, Norwegian Institute of Public Health, Oslo, Norway; 4 Norwegian Centre for Addiction Research, University of Oslo, Oslo, Norway

**Keywords:** Opioid substitution treatment, Drug prescriptions, Opioid analgesics, Treatment outcome, Chronic pain, Palliative care

## Abstract

**Background:**

Dispensations of opioid analgesics to patients on opioid agonist therapy (OAT) may increase the risk of overdoses. The current study’s objectives are to investigate the dispensation rates and mean daily doses of dispensed opioid analgesics among patients who received OAT opioids in Norway during 2013–2017 and evaluate whether discontinuing OAT opioids affects the dispensed dose of opioid analgesics.

**Methods:**

Information on opioids was collected from the Norwegian Prescription Database. Dispensation rates were calculated by dividing the number of patients who were dispensed at least one opioid analgesic by the number of patients who were dispensed an OAT opioid. We calculated the mean daily dose of opioid analgesics in oral morphine equivalents. The OAT opioid dose was defined as a ratio between the dispensed doses divided by the mean recommended dose. We used logistic regression to estimate the association between the dispensation of an opioid analgesic, a dose of OAT opioids, having chronic pain, and being on palliative care.

**Results:**

A total of 10,371 patients were dispensed at least one OAT opioid during the study period. In 2017, 18% were dispensed an opioid analgesic with a mean daily dose of 29 mg of oral morphine equivalents. Being dispensed an opioid analgesic was associated with having chronic pain (adjusted odds ratio (aOR): 3.6, 95% confidence interval: 3.2–4.2), being on palliative care (aOR: 6.1, 4.7–7.9), and receiving an OAT opioid dose below half of the recommended OAT dose (aOR: 1.7, 1.4–2.0). Similar results were seen in 2013–2016. The discontinuation of OAT opioids could increase the dose of dispensed opioid analgesics.

**Conclusion:**

Reducing the dispensation of opioid analgesics can be achieved by increasing the OAT opioid dose for patients on a low OAT dose, and by extending the period needed to taper off the OAT opioid dose at discontinuation.

## Background

At least 50% of all patients undergoing opioid agonist therapy (OAT) have somatic or mental comorbidities [1, 2], and more than 60% have physical comorbidities such as infections, and physical injuries that may require opioid analgesics (e.g. morphine, oxycodone, and ketobemidone) [2, 3]. High opioid tolerance and potentially significant individual differences in the dose needed to achieve pain relief make patients on OAT particularly challenging to treat adequately with opioid analgesics [4]. Therefore, co-dispensation, combining both opioid analgesics and OAT opioids (buprenorphine and methadone), are often required to suppress pain and treat opioid dependence on OAT. However, more than 20% percent of patients on OAT have psychiatric disorders such as attention deficit hyperactivity disorder [5], personality disorders [1], or psychosis diseases, and nearly 50% have other drug dependencies that may influence their ability to follow up the dispensed opioid analgesics as dosed [6, 7]. In these cases, concomitant dispensations of several opioids may potentially affect the risk of overdose deaths and intoxications, as well as aggravate pain if the opioids are not handled correctly [8–10].

Buprenorphine and methadone are the most frequently dispensed opioids in OAT worldwide. They are chosen due to their safety and long duration of action, and only one administration daily is needed [11–15]. Opioid analgesics are short and intermediate-acting agents that generally lack these properties, and they are thus not recommended for long-term therapy to support recovery from opioid dependence [8, 11, 16–18]. However, many OAT patients have chronic pain related comorbidities, and some receive palliative care, which may make the co-dispensations of opioid analgesics and OAT opioids essential [4, 19]. Balancing between achieving adequate pain relief and stabilizing the OAT rehabilitation is, in these contexts, challenging. Nevertheless, there has been limited research on dispensations of opioid analgesics to patients on OAT and the characteristics of patients dispensed opioid analgesics and OAT opioids concomitantly [20].

Norway experiences many of the same opioid-related complications as the United States, with high crude mortality rates related to opioid dependence and overdose [10, 21]. For a substantial proportion of these patients, OAT is an essential approach. In Norway, about 7500 patients received OAT opioids in 2017, and about 700 left treatment [22]. Of those who underwent OAT in the period 2007–2008, 12% were dispensed opioid analgesics [8], while the proportion of patients who left OAT and were dispensed opioid analgesics after discontinuation is unknown. By comparison, a recent Canadian study shows that 18% of patients who received methadone during OAT between 2003 and 2010 were dispensed opioid analgesics [9, 23]. In recent years, national programs in Norway have focused on cautious dispensation practices regarding opioid analgesics in order to reverse the opioid dependence epidemic [21, 24]. To complement this, more knowledge of the circumstances surrounding dispensed opioid analgesics in the OAT population is needed to be able to customize OAT for patients with severe opioid dependence.

Thus, this observational study aims to evaluate dispensed opioid analgesics among patients who were dispensed at least one OAT opioid (methadone or buprenorphine) in Norway between 2013 and 2017 by using national register-based data. More specifically, we will:

1. Calculate the dispensation rates and doses of dispensed opioid analgesics among patients who were dispensed at least one OAT opioid per calendar year.
2. Calculate, using logistic regression models per calendar year, whether the dispensation of an opioid analgesic was associated with age, gender, chronic pain, palliative care, and the number, type, and mean daily dose of dispensed OAT opioids among patients who were dispensed an OAT opioid.
3. Evaluate the dispensation rates and mean daily doses of dispensed opioid analgesics among patients who discontinued OAT.

## Methods

### Data source

All data used were national register data drawn from the Norwegian Prescription Database (NorPD) (www.norpd.no). From January 1, 2004, all pharmacies in Norway are obliged to submit all dispensed drug data electronically to the Norwegian Institute of Public Health. NorPD contains information concerning all drugs, including reimbursements, dispensed from pharmacies in Norway, except for dispensations that occur during hospitalization or at outpatient clinics. The Anatomical Therapeutic Chemical (ATC) classification system was employed according to determination by the World Health Organization (WHO) Collaborating Centre for Drug Statistics Methodology per October 2018 [25]. The NorPD data used in this study were collected from January 1, 2013 to March 31, 2018. The STROBE checklist was applied in preparation for the study (Additional file 1).

### Study population

We included all patients above 18 years of age who were dispensed at least one OAT opioid – including methadone, levomethadone, buprenorphine, and buprenorphine-naloxone – in the period January 1, 2013 to December 31, 2017. Patients who were dispensed methadone tablets to achieve pain relief in palliative care were excluded. These were identified in the data as those who were dispensed methadone tablets without any dispensations of other OAT opioids or methadone mixtures during the period from January 1, 2004, to December 31, 2017. We defined discontinuation of OAT as any patient who had their last dispensation of an OAT opioid within the inclusion period January 1, 2017 to September 30, 2017, with no further dispensations until the end of the NorPD data collection period on March 31, 2018. Any patient who died was excluded from the calendar year of their death.

### Analysis strategy and statistical analyses

#### Definitions of OAT opioids, opioid analgesics, age, chronic pain, palliative care, type and number of dispensations, and doses of OAT opioid and dispensed opioid analgesics

During the study, we defined opioid agonist therapy opioids and opioid analgesics according to their ATC codes (Additional file 2) and categorized all patients into age groups: ≤ 25, 26–35, 36–45, 46–55, and ≥ 56. We defined ‘patients with chronic pain’ as those who were dispensed drugs during the study period for reimbursement due to chronic pain. Similarly, ‘patients on palliative care’ were defined as all patients who were dispensed drugs for reimbursement due to palliative care between 2013 and 2017. Patients who were dispensed drugs for reimbursement concerning both chronic pain and palliative care were categorized as ‘patients on palliative care’. Moreover, the type of OAT opioid was defined as the last type dispensed per calendar year. The number of dispensations of an opioid was defined as all dispensations of a drug. Furthermore, we calculated the doses of dispensed opioid analgesics and OAT opioids by using the daily defined dose (DDD) stated in NorPD and defined by the WHOs standards (Additional file 3) [25]. For opioid analgesics, we summarized all dispensed DDD per opioid per calendar year and converted the total DDDs into milligrams. Further, we calculated all the dispensed milligrams per opioid and converted them to oral morphine equivalents (OMEQ) based on equipotency [26]. The total sum of all dispensed morphine equivalents per patient was summarized per year and divided by 365.25 days. For OAT opioids, we calculated an OAT opioid dose ratio based on the mean dispensed OAT opioid dose per day and divided by the mean recommended dose for OAT per day. We defined a mean recommended OAT opioid dose as the mean dose needed to support the recovery from the opioid dependence per day as defined by WHOs recommendations: 90 mg methadone, 45 mg levomethadone, 18 mg buprenorphine, or 18/4.5 mg buprenorphine-naloxone [27]. The dispensed OAT opioid dose per day was calculated by summarizing all DDD of each OAT opioid per year. The OAT opioids were converted from DDD to milligrams according to the WHOs standard [25]. Further, the milligrams were divided by 365.25 days and the mean recommended dose per day. Finally, the OAT opioid dose ratio from each OAT opioid was summarized to the OAT opioid dose ratio.

The OAT opioids were also categorized into four dispensation groups per year: 1–6 (less than every second month), 7–12 (less than monthly), 13–51 (at least monthly), and ≥ 52 (at least weekly). For the dispensed doses of OAT opioids, patients were categorized into three groups based on the OAT opioid dose ratio: < 0.5, 0.5–1, and > 1. Patients who were dispensed an OAT opioid dose > 1 indicated a daily dose above the mean dose during OAT.

#### Analysis strategy according to the objectives

Dispensation rates were defined as all patients on OAT who were dispensed at least one opioid analgesic per year divided by all included patients who were dispensed an OAT opioid the same year. We divided opioid analgesics into two drug groups based on their potency when presenting the dispensation rates: weak opioid analgesics (codeine, tramadol, and tapentadol) and strong opioid analgesics (all opioid analgesics apart from codeine, tramadol, and tapentadol). We presented the total dispensed doses of opioid analgesics as a mean daily dose per patient per year stated in OMEQ.

Using logistic regression models, we evaluated the association between being dispensed an opioid analgesic and the number and type of dispensed OAT opioids, as well as the OAT opioid dose ratio, the patient’s age and gender, and whether they had chronic pain or were on palliative care. The models were run with all included patients per year and those who had 13 or more dispensations of OAT opioids per year. Concerning the latter population, we investigated whether the associations were consistent for those who frequently dispensed OAT opioids when compared with including all patients.

All patients who discontinued OAT were identified. For these patients, the dispensations of opioid analgesics were categorized into three periods: 180–90 days before, 90 days before, and 90 days after the discontinuation date. The interval 180 to 90 days before the discontinuation date was defined as the baseline. The dispensation rates were defined as all patients who dispensed an opioid analgesic in the periods divided by the number of patients who discontinued OAT. The dispensed doses of opioid analgesics per day were calculated by dividing the number of dispensed OMEQ for each period by 90 days. The calculation of OMEQ dose was similar to when calculating the OMEQ dose per year. The mean OMEQ dose before and after discontinuation were compared.

### Statistical analyses

The means, median, percentiles, percentage, 95% confidence interval (CI), odds ratio (OR), and *p*-value are presented. The paired t-test was used to compare the mean doses of dispensed opioid analgesics before and after the discontinuation date of OAT. Multivariate analyses for categorical variables were performed per year by creating logistic regression models. In these models, the dependent categorical variable was being dispensed an opioid analgesic during a calendar year. Age groups, gender, having chronic pain, being on palliative care, the type of OAT opioid, the number of dispensed OAT opioids, and the OAT opioid dose ratio were handled as independent and categorical variables. The level of statistical significance was set to *p* < 0.05. SPSS version 24 was used for all statistical analyses.

### Ethical considerations

The Regional Committee for Medical and Health Research Ethics, REC vest, Norway, approved the use of registry data for the study (approval number 2018/939/REK Vest, June 19, 2018). No informed consent from included patients was necessary.

## Results

### Basic characteristics

A total of 10,371 patients were dispensed at least one OAT opioid from pharmacies in Norway in the period from 2013 to 2017. In 2017, 69% were men, and the mean age was 45 years. Buprenorphine or buprenorphine-naloxone was dispensed to 61% of the patients (Table 1). During the study period, 737 patients were dispensed at least one reimbursed drug due to palliative care, and 2011 patients received at least one reimbursed drug due to chronic pain. A total of 692 patients died during the study period.

**Table 1:** Basic characteristics of patients who were dispensed an OAT opioid

Basic characteristics	2013	2014	2015	2016	2017
	*No. (%)*	*No. (%)*	*No. (%)*	*No. (%)*	*No. (%)*
Patients	7709	7914	7958	7804	7709
Deaths	165	151	138	114	124
*Patients, excluded deaths*	*7544*	*7763*	*7820*	*7690*	*7585*
*Age*					
≤ 25	211 (3)	185 (2)	171 (2)	135 (2)	120 (2)
26–35	1590 (21)	1570 (20)	1551 (20)	1403 (18)	1333 (18)
36–45	2724 (36)	2730 (35)	2605 (33)	2508 (33)	3292 (32)
46–55	2283 (30)	2449 (32)	2544 (33)	2540 (33)	2548 (34)
≥ 56	736 (10)	829 (11)	949 (12)	1104 (14)	1192 (16)
*Mean (SD)*	*43 (10)*	*44 (10)*	*44 (10)*	*44 (10)*	*45 (10)*
*Gender*					
Male	5221 (69)	5390 (69)	5430 (69)	5354 (70)	5245 (69)
Female	2323 (31)	2373 (31)	2390 (31)	2336 (30)	2340 (31)
*OAT opioids*^a^					
Methadone (included levomethadone)	3406 (45)	3264 (42)	3216 (41)	3066 (40)	2981 (39)
Buprenorphine (included combination with naloxone)	4138 (55)	4499 (58)	4604 (59)	4624 (60)	4604 (61)
*Dispensed opioid analgesics*					
- All	1430 (19)	1424 (18)	1400 (18)	1382 (18)	1359 (18)
*- Strong opioid analgesic*	*542 (7)*	*518 (7)*	*490 (6)*	*534 (7)*	*526 (7)*
- W*eak opioid analgesic*	*1089 (14)*	*1078 (14)*	*1066 (14)*	*1011 (13)*	*997 (13)*
Dose of dispensed opioid analgesics (in OMEQ)					
- mean (mg/year)	18,538	17,399	8963	9361	10,723
- mean (mg/day/year)	51	48	25	26	29
- median (mg/year)	450	400	400	500	500
- median (mg/day/year)	1	1	1	1	1
- 25 percentile (mg/year)	86	75	90	100	100
- 25 percentile (mg/day/year)	0	0	0	0	0
- 75 percentile (mg/year)	4478	3321	2616	2891	3500
- 75 percentile (mg/day/year)	12	9	7	8	10
*Patients on palliative care*^b^	737				
*Patients with chronic pain*^c^	2011				

*No.: Number of patients; OAT* Opioid agonist therapy, *OMEQ* Oral morphine equivalents, *SD* Standard deviation

^a^ The type of OAT opioid that was dispensed on the last dispensation per year

^b^ Patients who were dispensed drugs that were reimbursed due to palliative care in the study period

^c^ Patients who were dispensed a drug that was reimbursed due to chronic pain in the study period. Patients who were dispensed drugs that were reimbursed for palliative care and chronic pain were classified as ‘patients on palliative care’

### Dispensation rates and mean daily doses of opioid analgesics

The proportion of OAT patients who were dispensed at least one weak or strong opioid analgesic was 18% in 2017. In the same year, 13% were dispensed at least one weak opioid analgesic, and 7% were dispensed at least one strong opioid analgesic. The most dispensed weak opioid analgesic was codeine (9% in 2017), and the most dispensed strong opioid analgesic was oxycodone (3% in 2017) (Table 2). Similar results were seen in the period 2013–2016. The mean daily dose of dispensed opioid analgesics was 51 mg OMEQ per day in 2013 and 29 mg OMEQ per day in 2017.

**Table 2:** The proportion of patients who were dispensed an opioid analgesic

Year	2013	2014	2015	2016	2017
Opioid analgesics	No. (%*)	No. (%*)	No. (%*)	No. (%*)	No. (%*)
Number of patients	7544	7763	7820	7690	7585
*Strong opioid analgesics*					
Oxycodone	234 (3)	226 (3)	205 (3)	231 (3)	246 (3)
Morphine	184 (2)	143 (2)	120 (2)	134 (2)	149 (2)
Fentanyl	79 (1)	58 (1)	39 (0)	11 (0)	34 (0)
Buprenophine (non OAT)	129 (2)	138 (2)	154 (2)	163 (2)	145 (2)
Ketobemidone	51 (1)	55 (1)	48 (1)	49 (1)	31 (0)
Pethidine	6 (0)	6 (0)	5 (0)	< 5 (0)	5 (0)
Hydromorphone	12 (0)	5 (0)	< 5 (0)	< 5 (0)	< 5 (0)
*Weak opioid analgesics*					
Codeine	822 (11)	799 (10)	781 (10)	730 (9)	690 (9)
Tramadol	392 (5)	397 (5)	398 (5)	375 (5)	417 (5)
Tapentadol	14 (0)	7 (0)	6 (0)	10 (0)	10 (0)

The table displays the proportion of patients on OAT who were dispensed a weak and a strong opioid analgesics in the period 2013–2017. We only included opioid analgesics that had marketing authorization in Norway in the study period

*No*.: Number of patients, *OAT* Opioid agonist therapy

* Percent of patients who were dispensed an OAT opioid

### Factors associated with being dispensed an opioid analgesic

We found that being dispensed an opioid analgesic was associated with being female, on palliative care, having chronic pain, and dispensed OAT opioids less than every second month (≤ 6 dispensations per year), compared with being dispensed OAT opioids at least weekly (≥ 52 dispensations per year) when including all patients in 2017 (Table 3). In addition, we found that being dispensed an opioid analgesic was associated with having an OAT opioid dose ratio below 0.5 compared with an OAT opioid dose ratio above 1.0. Similar results were seen in the period 2013–2016 (Additional file 4). For patients who were dispensed OAT opioids at least monthly (13 or more dispensations per year), we found that the independent variables (gender, age group, having chronic pain, being on palliative care, the type of dispensed OAT opioids, and OAT opioid dose ratio) were substantially unchanged when including all patients (Additional file 5).

**Table 3:** Logistic regression of factors associated with being dispensed an opioid analgesic in 2017

Patients who had ≥1 dispensation of an opioid analgesic	*N* = 1359		
	cOR	aOR (95% CI)	***p***-value
Age			
≤ 25	1.00 (ref.)	1.00 (ref.)	
26–35	0.93	1.01 (0.60–1.70)	.964
36–45	0.90	1.00 (0.60–1.66)	.985
46–55	1.17	1.31 (0.78–2.18)	.304
≥ 56	1.55	1.59 (0.95–2.68)	.080
*Gender*			
Male	1.00 (ref.)	1.00 (ref.)	
Female	*1.64*	*1.44 (1.27–1.64)*	*< .001*
*Patients on palliative care*	*5.00*	*6.08 (4.67–7.92)*	*< .001*
*Patients with chronic pain*	*3.30*	*3.64 (3.16–4.19)*	*< .001*
*The number of dispensations of OAT opioids per year*			
≥ 52 (at least weekly)	1.00 (ref.)	1.00 (ref.)	
13–51 (at least monthly)	1.10	1.10 (0.88–1.37)	.423
7–12 (less than monthly)	1.35	1.10 (0.85–1.43)	.454
1–6 (less than every second month)	*1.98*	*1.44 (1.07–1.93)*	*.016*
*OAT opioids*^a^			
Buprenorphine/Buprenoprhine-naloxone	1.00 (ref.)	1.00 (ref.)	
Methadone/Levomethadone	1.04	0.96 (0.84–1.10)	.542
*OAT opioid dose ratio per day*^b^			
> 1	1.00 (ref.)	1.00 (ref.)	
0.5–1	0.85	0.95 (0.80–1.11)	.497
< 0.5	*1.61*	*1.69 (1.41–2.03)*	*< .001*

The table displays the association between being dispensed an opioid analgesic and age groups, gender, palliative care, chronic pain, the number of dispensations of OAT opioids, the type of dispensed OAT opioid, and the OAT opioid dose ratio among all patients who were dispensed an OAT opioid in 2017

*aOR* adjusted odds ratio, *cOR* crude odds ratio, *CI* confidence interval, *OAT* opioid agonist therapy

^a^ The type of OAT opioid that was dispensed on the last dispensation

^b^ We defined the OAT opioid dose ratio as a mean daily dose of dispensed OAT opioids divided by mean recommended daily dose of OAT opioids (18 mg buprenorphine or 18/4.5 mg buprenorphine/naloxone, 90 mg methadone or 45 mg levomethadone). A ratio of one indicated that patients were dispensed a mean daily dose of OAT opioids equal to the mean recommended dose per day

### The dispensation rates and mean daily doses of opioid analgesics among patients who discontinued OAT

A total of 693 patients discontinued OAT during the period January 1, 2017 to September 30, 2017. Of these, 111 (16%) patients were dispensed an opioid analgesic in the last 180 days before the discontinuation date (Table 4). In the 90 days following the discontinuation date, 122 (18%) were dispensed an opioid analgesic. Patients who were only dispensed opioid analgesics in the last 90 days before the discontinuation date (not at baseline) increased the mean daily dose of dispensed opioid analgesic from 38 mg OMEQ to 347 mg OMEQ in the 90 days following the discontinuation date (Δ mean 308, 95% CI: 22–594, *p* = 0.04). For patients who only were dispensed an opioid analgesic in the 90 days after the discontinuation date, the mean dose of opioid analgesic was 91 mg OMEQ per day.

**Table 4:**
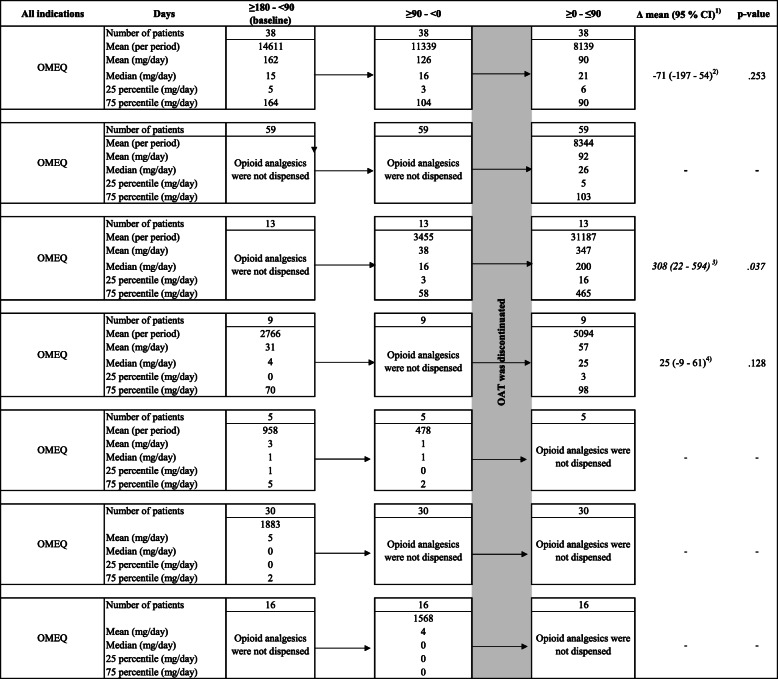
Dispensation of opioid anagetics among patients who discontinued OAT

The table displays the mean doses of dispensed opioids analgesics among patients who discontinued OAT. Patients’ opioid dispensations were analyzed in three periods according to the OAT disconatinuation date: 180–90 days before, 90 days before, and 90 days after the discontinuation date. All doses of dispensed opioid analgesics were summarized in each period and converted to OMEQ. The mean, median, 25 percentile and 75 percentile were calculated for the three periods. For those who were dispensed opioid analgesics in the periods before and after the discontinuation date, we have used a paired t-test for calculating differences in dispensed doses of the opioid analgesics.

*CI* Confidence interval, *Df* Degree of freedom, *OAT* Opioid agonist therapy, *OMEQ* Oral morphine equivalents.

^1)^ The change in opioid analgesic dose when comparing dispensed doses before discontinuation with dispensed doses after discontinuation

^2)^ Paired t-test of the mean daily dose at the 90 days after discontinuation compared with the mean daily dose at baseline (180 to 90 days before discontinuation). Df = 37

^3)^ Paired t-test of the mean daily dose at the last 90 days before discontinuation compared with the mean daily dose at the 90 days after discontinuation. Df = 12

^4)^ Paired t-test of the mean daily dose at the 90 days after discontinuation compared with the mean daily dose at baseline (180 to 90 days before discontinuation). Df = 8

## Discussion

The proportion of patients on OAT in Norway who were dispensed an opioid analgesic in the study period was 18% yearly. By comparison, 11% were dispensed an opioid analgesic in the general Norwegian population in 2017 [28]. The proportion of OAT patients who were dispensed weak opioid analgesics was higher than those dispensed strong opioid analgesics. Oxycodone was the most dispensed strong opioid analgesic, and codeine was the most dispensed weak opioid analgesic. The dispensation of an opioid analgesic was associated with being: female, on palliative care, having chronic pain, dispensed OAT opioids less than every second month compared with no less than weekly dispensations, and dispensed an OAT opioid dose below half the recommended mean dose compared with a dose above the mean recommended dose. Discontinuation of OAT did not substantially affect the proportion of patients who were dispensed opioid analgesic. However, doses of dispensed opioid analgesics were higher among those who initiated opioid analgesics the last 90 days before discontinuation.

18% of patients on OAT were dispensed at least one opioid analgesic in 2017, which was an increase from 12% compared with the period from 2007 to 2008 in the OAT population [8]. The increase of dispensed opioid analgesics may be related to changes in the Norwegian eligibility criteria that grants OAT. During recent years, patients with opioid dependence using street drugs besides opioids have been offering OAT rehabilitation in terms of increasing the coverage of OAT rehabilitation and reducing opioid overdoses and complications associated with injecting street opioids [22, 29, 30]. In addition, patients with chronic pain who develop an opioid dependence through long-term dispensations of opioid analgesics are offered OAT if opioid discontinuation is unsuccessful. By granting OAT to a broader specter of patients with opioid dependence, the OAT population may have become more physical and psychiatrically comorbid, which may explain the increase of dispensed opioid analgesics during the last decade [22]. However, the dispensation rates of opioid analgesics in the Norwegian OAT population were substantially low compared with studies on the OAT population in Canada, which shows a prevalence of dispensed opioid analgesics between 18 and 34% [9, 23].

An OAT opioid dose below half the recommended mean dose was associated with being dispensed an opioid analgesic. This finding may point out that a low dose of OAT opioids does not adequately protect against craving and opioid withdrawal, causing concomitant dispensations of opioid analgesics. Kurdyak et al. [23] assessed the dispensations of opioid analgesics in an OAT population. The authors found that opioid analgesics were more often dispensed by prescribers that were not responsible for OAT rehabilitation [23]. A lack of optimal OAT dose may be a possible explanation for why patients seek other prescribers to be dispensed opioid analgesics. This is of particular importance to ensure an adequate OAT opioid dose to stabilize opioid dependence and prevent dispensations of opioid analgesics to treat opioid withdrawal and cravings. Therefore, guidelines preferably recommend the long-acting opioids methadone or buprenorphine during OAT rehabilitation [29, 31, 32].

Patients on OAT that have chronic pain or on palliative care are associated with being dispensed opioid analgesics. The results are consistent with results seen in a comparable study on OAT patients [9] and the overall Norwegian population [33]. In general, there is a lack of knowledge showing a persistent analgesic effect of long-term use of opioid analgesics on chronic pain [34]. A recent study showed that OAT patients with chronic pain were associated with chronic hepatitis C virus infection, mental health disorder, low-income status, and alcohol dependence compared with the general OAT population [35]. These medical and psychosocial comorbidities may influence and exacerbate the patient’s subjective experience of pain, making the pain management with opioid analgesics particularly challenging [36, 37]. On the other hand, OAT patients on palliative care can improve quality of life if adequate management of pain and opioid dependence is provided [38]. A close collaboration between health professionals in several health and social care teams can be the key to success among patients with chronic pain and those on palliative care, particularly if there is an ongoing use of illegal drugs. Handling opioid analgesics for these patients with complex medical and psychosocial conditions is not discussed in the current worldwide OAT guidelines [39–41]. Nevertheless, our findings point out that the proportion of Norwegian OAT patients above 56 years increased from 10 to 16% from 2013 to 2017. This increase in elderly OAT patients may predict a need in the next coming decades for guidelines on how to handle chronic pain and palliative care for such patients. In addition, this emphasizes the importance of customizing the OAT for ensuring further sufficient opioid agonist therapy and adequate pain management in this population [42–44].

Discontinuing OAT did not substantially change the number of patients who were dispensed opioid analgesics. However, initiating dispensations of opioid analgesics during the 90 days prior to the discontinuation date increased the doses of dispensed opioid analgesics significantly when compared with the dispensed doses during the 90 days following the discontinuation date. For these patients, one can assume that opioid analgesics were typically dispensed to suppress opioid withdrawal and craving due to the OAT opioid dose’s rapid tapering off. Planning an OAT opioid dose’s long-term tapering off at discontinuation is essential to prevent increased mortality, should the patient relapse to street opioids, and limit dispensations of opioid analgesics to treat opioid withdrawal [14].

### Strengths and limitations

The use of national registry data has some clear strengths, such as capturing whole cohorts of the studied populations. Pharmacy records are considered more valid than both medical records and data collected from questionnaires and interviews. Because practically all dispensed drugs are registered in the NorPD database, the completeness and precision of all received information is high, and the potential for information bias is low.

This study does however have some limitations. First, about 10% of the OAT patients were not registered by NorPD. These patients received OAT opioids from outpatient clinics in primary health care or specialized health care. Some of these outpatient clinics ordered OAT opioids directly from pharmacies without any linkage to a personal identification number. These patients were lost in this study [22]. Second, the annual self-reporting survey of OAT suggested that the mean number of dispensations of OAT opioids were four times a week [22], which did not wholly match our estimates. To adjust for the uncertainty concerning which was most correct, we used the OAT opioid dose ratio measuring the mean recommended daily dose to complement our results. Third, the NorPD only receives information about dispensed drugs, and we could not know whether the drugs have been consumed or whether the patients were using illegal drugs concomitantly. Fourth, the dispensations of opioid analgesics may have confounders. Therefore, we adjusted for two potentially strong confounders by identifying patients who were dispensed drugs on reimbursement codes for chronic pain and palliative care in the database. Nevertheless, a minor part of the medical indications for dispensed drugs was available by using reimbursement codes in NorPD [28].

## Conclusion

18% of Norwegian OAT patients per year were dispensed an opioid analgesic in the period 2013–2017. A substantial proportion of those who were dispensed opioid analgesics had an OAT opioid dose less than half the recommended mean dose, were on palliative care, or had chronic pain. In addition, discontinuing OAT increased the doses of dispensed opioid analgesics among patients that initiated opioid analgesics the last 90 days before discontinuation. A reduction in the proportion of OAT patients dispensed opioid analgesics may be achieved by increasing the OAT opioid dose for patients taking low-dose OAT opioids and facilitating it for those with chronic pain and on palliative care. For patients who discontinue OAT, an extended period of tapering off can reduce the dispensations of opioid analgesics after discontinuation.

## Supplementary information

**Additional file 1.** STROBE Statement. Checklist of items included in reports of cohort studies.

**Additional file 2.** Included opioids. ATC: Anatomical Therapeutic Chemical. All included opioids with ATC codes categorized into opioid analgesics and opioid agonist therapy opioids.

**Additional file 3. **Defined daily doses (DDD) of OAT opioids and opioid analgesics converted to milligrams and morphine equivalents. DDD: Defined daily doses; OMEQ: oral morphine equivalents. The table displays the conversion of defined daily doses (DDD) to milligrams according to the WHOs’ standards (1) and the calculations of opioid analgesics to morphine equivalents (2). Only opioid analgesics that had marketing authorizations in Norway in the study period were included. (1) **Definition and general considerations**. In: *https://www.whocc.no/ddd/ definition_and_general_considera/#Definition**.* WHO Collaborating Centre for Drug Statistics; 2019.(2) Svendsen K, Borchgrevink P, Fredheim O, Hamunen K, Mellbye A, Dale O: Choosing the unit of measurement counts: the use of oral morphine equivalents in studies of opioid consumption is a useful addition to defined daily doses. *Palliat Med* 2011, **25**(7):725–732.

**Additional file 4.** Logistic regression of factors associated with being dispensed an opioid analgesic OAT = opioid agonist therapy; cOR = crude odds ratio; aOR = adjusted odds ratio; CI = confidence interval. * The type of OAT opioid that was dispensed on the last dispensation per year ** We defined the OAT opioid dose ratio as a mean daily dose of dispensed OAT opioids divided by mean recommended daily dose of OAT opioids (18 mg buprenorphine or 18/4.5 mg buprenorphine/naloxone, 90 mg methadone or 45 mg levomethadone). A ratio of one indicated that patients were dispensed a mean daily dose of OAT opioids equal to the mean recommended dose per day. The table displays the association between being dispensed an opioid analgesic and age groups, gender, palliative care, chronic pain, the number of dispensations of OAT opioids, the type of dispensed OAT opioid, and the OAT opioid dose ratio among all patients who were dispensed an OAT opioid in the period 2013–2016.

**Additional file 5.** Logistic regression of factors associated with being dispensed an opioid analgesic. OAT: opioid agonist therapy; cOR: crude odds ratio; aOR: adjusted odds ratio; CI: confidence interval. * The type of OAT opioid that was dispensed on the last dispensation per year. ** We defined the OAT opioid dose ratio as a mean daily dose of dispensed OAT opioids divided by mean recommended dose of OAT opioids per day (18 mg buprenorphine or 18/4.5 mg buprenorphine/naloxone, 90 mg methadone or 45 mg levomethadone). A ratio of one indicated that patients were dispensed a mean daily dose of OAT opioids equal to the mean recommended dose per day. The table displays the association between being dispensed an opioid analgesic and age groups, gender, palliative care, chronic pain, the number of dispensations of OAT opioids, the type of dispensed OAT opioid, and the OAT opioid dose ratio among patients who were dispensed an OAT opioid at least monthly per year in the period 2013–2017.

## Data Availability

Except supplemental tables with some additional data, no additional data are available due to data protection requirements.

## Abbreviations

AOR: Adjusted odds ratio
ATC: Anatomical Therapeutic Chemical
CI: Confidence interval
DDD: Defined Daily Doses
HELFO: The Norwegian Health Economics Administration
NorPD: The Norwegian Prescription Database
OAT: Opioid agonist therapy
OMEC: Oral morphine equivalent
OR: Odds ratio
REC: The Regional Committee for Medical and Health Research Ethics
WHO: World Health Organization
